# The physical foundation of *F*_N_ = *kh* ^3/2^ for conical/pyramidal indentation loading curves

**DOI:** 10.1002/sca.21223

**Published:** 2015-05-15

**Authors:** G. Kaupp

**Affiliations:** ^1^ University of Oldenburg, Faculty 5EdewechtGermany

**Keywords:** force‐depth relation, nanoindentation loading curves, penetration resistance, physical deduction, pressure and plasticizing

## Abstract

A physical deduction of the *F*_N_ = *kh* ^3/2^ relation (where *F*_N_ is normal force, *k* penetration resistance, and *h* penetration depth) for conical/pyramidal indentation loading curves has been achieved on the basis of elementary mathematics. The indentation process couples the productions of volume and pressure to the displaced material that often partly plasticizes due to such pressure. As the pressure/plasticizing depends on the indenter volume, it follows that *F*_N _= *F*_Np_^1/3^  ·  *F*_NV_^2/3^, where the index p stands for pressure/plasticizing and V for indentation volume. *F*_Np_ does not contribute to the penetration, only *F*_NV_. The exponent 2/3 on *F*_NV_ shows that while *F*_N_ is experimentally applied; only *F*_N_^2/3^ is responsible for the penetration depth *h*. Thus, *F*_N _= *kh* ^3/2^ is deduced and the physical reason is the loss of *F*_N_^1/3^ for the depth. Unfortunately, this has not been considered in teaching, textbooks, and the previous deduction of numerous common mechanical parameters, when the Love/Sneddon deductions of an exponent 2 on *h* were accepted and applied. The various unexpected experimental verifications and applications of the correct exponent 3/2 are mentioned and cited. Undue mechanical parameters require correction not only for safety reasons. SCANNING 38:177–179, 2016. © 2015 The Authors. *Scanning* published by Wiley Periodicals, Inc.

## Introduction

In 1939, Love and in 1965, Sneddon mathematically solved the “Boussinesq's problem” with different results, but both with the prediction of normal force (*F*_N_) being proportional to depth square for conical and, thus, also pyramidal indentations. This has been widely accepted in publications and leading textbooks, and used for the deduction of various mechanical parameters that are still in use. Exponent 2 is also the result of numerous finite element simulations, when these use quadratic displacement elements (for example Wang *et al*., 2008; cf. Soare *et al*., 2005). Such simulations are often claimed to concur with published loading curves. However, more precise analysis reveals since 2004 that the experimental exponent is 3/2 instead. Simulated and experimental curves do not even correspond when published in the same paper. Only the analysis using the correct exponent can show, how to distinguish initial surface effects and phase changes under the load if these occur (Kaupp and Naimi‐Jamal, 2004; Naimi‐Jamal and Kaupp, 2005). The linear correlation coefficient for the slope *k* (from *F*_N_ vs. *h* ^3/2^) continues to always prove r > 0.999 or for less noisy measurements r > 0.9999 (Kaupp and Naimi‐Jamal, 2010, and the cited more recent publications up to 2014). It was, therefore, possible to introduce the concept of penetration resistance (*k*) for the safe comparison of materials' properties and compatibilities (Kaupp and Naimi‐Jamal, 2013), the energetic of indentations with the important finding that 80% of *F*_N_ is used for the indentation work and 20% for all the other force‐induced energetic events (Kaupp, 2013). Temperature dependent indentations even allow for the calculation of the activation energy of phase changes from nothing else than from indentation loading curves (Kaupp, 2014). What's still missing was the physical reason for the experimentally verified successful exponent 3/2 on *h*, and this has been rightfully asked for by new‐comers and experts in the field. Thus, the appreciation of the new exponent against textbooks (except Kaupp, 2006) requires the deduction of the Title formula. We report now on an unexpectedly short deduction of the physical reason that was not thought upon till now.

## Experimental Background

The instrumental indentation experiment uses in most cases a diamond indenter that is continuously pressed with normal force (*F*_N_) onto a level surface until the continuously recorded depth *h* is reached. By doing so, the volume *V* of the indenter is intruded and it shifts material towards the bulk while producing pressure to it. Depending on the materials' properties, such pressure *p* may persist (fully elastic) or it is partly released by some sort of plasticizing and migration with all of the known long‐range effects. This scheme is principally equivalent with all of the different loading types normal to level surfaces and has been experimentally verified for all mechanisms of plasticizing (Kaupp, 2011; Kaupp and Naimi‐Jamal, 2013). Such retained pressure is, of course, used in unloading curves for the calculation of the elastic modulus, which does, however, not apply to the present topic. With this in mind, we can start the deduction of the exponent 3/2.

## Results and Discussion

The indentation couples two processes that must be differentiated because the applied force must serve both of them. The production of volume is attributed to the fraction *F*_NV_^m^ for indentation. The production of pressure + loss of pressure (loss by plasticizing via pressure) to the displaced material is attributed to the fraction *F*_Np_^n^ for pressure. As the multiplication of both factors must give the product *F*_N_, these fractional forces must have the exponents m and n < 1, so that we obtain Equation (1).

(1) $$F_{N}={F_{Nv}}^{m}\cdot {F_{Np}}^{n}$$

For the determination of the exponents m and n, we use the total pressure that could be reached at the depth *h* for absence of plasticizing. It is (*p* + loss of *p*) and we call it *p*_total_. Equation (2) is evident, and the mathematical expression for the cone volume is *V*_cone_.

(2) $$p_{total}=KV;V_{cone}=\pi {\left(\tan\alpha \right)}^{2}h^{3}/3$$

Equation (2) reveals that *p*_total_ and, thus, also *F*_Np_ are proportional to *h* ^3^ of the immersed cone. Formula (3) is, thus, obtained for cones and pyramids (with their “effective cone angles” α).

(3) $$p_{total}\propto h^{3}andthusalsoF_{Np}\propto h^{3}$$

Formula (3) reveals the *F*_Np_^1/3^ proportionality to the depth *h*, but *F*_Np_^1/3^ does not contribute to the depth. Nevertheless, when n = 1/3, m must be 2/3 according to Equation (1), and this gives Equation (4).

(4) $$F_{N}={F_{NV}}^{2/3}\cdot {F_{Np}}^{1/3}$$

The exponent 2/3 on *F*_NV_^2/3^ in Equation (4) reveals that while the instrumental indentation applies *F*_N_, only the fraction *F*_NV_^2/3^ is responsible for the penetration and its depth *h*. This is expressed with the searched‐for Equation (5), where we do no longer need the index V.

(5) $${F_{N}}^{2/3}\propto horF_{N}=kh^{3/2}$$

The unavoidable pressure/plasticizing factor *F*_Np_^1/3^ is lost for the depth. This is the physical reason for the exponent 3/2 on *h* instead of recently assumed 2 for cones and pyramids.

## Conclusions

The straightforward physical deduction of the exponent 3/2 on *h* with elementary mathematics for indentation loading curves of cones and pyramids reveals a clear‐cut physical reason. It will certainly strengthen the appreciation of exactly quantitative instrumental nano‐, micro‐, and macro‐indentations with conical/pyramidal indenters. When required, the respective penetration resistance constant *k* (N/m^3/2^) can be easily parameterized (see Equation (2)). An example would be when a penetration resistance *k* shall be compared with different indenter half‐angles α. But when the exponent on *h* of loading curves is used for hardness *H*, modulus *E*, or further parameter calculations, the correct exponent 3/2 should be used (but not 2 as for example at Oliver, 2001, and many others). Also the numerous recent plasticity parameters for biological materials in a tutorial of Oyen and Cook (2009) were deduced with the unsupported exponent 2 on *h*, and require urgent correction. Only the correct exponent 3/2 allows for more advanced important applications that revealed and will reveal unexpected materials' qualities. Some of these are named in the Introduction, others can be found in the cited papers of the present author. Reliable mechanical qualities on the sound physical basis are most important for the proper adjustment of technical and medicinal composites and joints, for safety reasons. This is particularly important in the pressure range for phase changes, the onset of which can only be detected in the loading curves by analysis with the correct exponent 3/2 on *h*. It is hoped that all of that will now be acknowledged in teaching, textbooks, and used for technical applications.
